# Short- and long-term outcomes of systemic semilunar valve replacement in neonates and infants

**DOI:** 10.1038/s44325-026-00109-6

**Published:** 2026-03-23

**Authors:** Abdelrahman Masri, Caroline Yunhua Shi, Brent Winemiller, Haya Alsarrawi, Lazaros K. Kochilas, Brian Reemtsen, Amna Qasim, Taufiek Konrad Rajab

**Affiliations:** 1 Department of Pediatrics, Division of Pediatric Cardiology, Arkansas Children’s Hospital, Little Rock, AR USA; 2 Department of Pediatrics, Emory University School of Medicine, Atlanta, GA USA; 3 Department of Pediatrics, Arkansas Children’s Hospital, Little Rock, AR USA; 4 Department of Pediatrics, Emory University School of Medicine, Atlanta, Georgia, USA; Children’s Healthcare of Atlanta Cardiology, Atlanta, GA USA; 5 Department of Cardiothoracic Surgery, Arkansas Children’s Hospital, Little Rock, AR USA

**Keywords:** Cardiology, Diseases, Health care, Medical research

## Abstract

Systemic semilunar valve replacement in neonates and infants is rare and usually a last resort. We analyzed Pediatric Cardiac Care Consortium data for patients undergoing Ross, aortic valve replacement (AVR), or truncal valve replacement (TVR) from 1982–2011 across 35 centers, with mortality tracked via the US National Death Index through 2022. Among 167 patients, in-hospital mortality was 23% for Ross, 49% for AVR, and 52% for TVR. Twenty-five–year survival was 59%, 29%, and 41%, respectively. Neonatal age (vs. infant) was associated with increased in-hospital and long-term mortality (OR 2.5, 3.9, respectively), while higher surgical weight was protective (OR 0.67, 0.61 per kg, respectively). The earlier surgical era was associated with higher in-hospital mortality (OR 3.4). AVR had over threefold in-hospital and long-term mortality (OR 3.2, 3.4, respectively). These results highlight the historically high risk of systemic semilunar valve replacement in this population and the need for innovative surgical approaches.

## Introduction

Systemic outflow abnormalities account for a small portion of congenital heart disease (CHD)^1^, with an incidence of 401 per million for significant aortic valve stenosis (prevalence 5.4% of all CHD), and 107 per million for truncus arteriosus (prevalence 0.7% of all CHD)^1^. While repair is preferred when feasible in neonates and infants to preserve native tissue, support growth, and delay timing of replacement, semilunar valve replacement becomes necessary when repair fails or is not deemed feasible, with options for replacement including prosthetic valves, autografts, and allografts^2–5^.

Aortic valve replacement (AVR) using homograft valves in neonates and infants is associated with high early mortality, reported at 40% in a 2012 STS database study^6^. To our knowledge, bioprosthetic AVR has not been reported in infants; however, rapid structural degeneration is noted in older children^7^. Mechanical valve replacements have been described in infants undergoing truncal valve replacement (TVR)^8^, but not AVR, and requires lifelong anticoagulation.

The Ross procedure (Ross), which uses the patient’s pulmonary valve^8^, has lower mortality (9% for Ross and 19% for Ross-Konno^6^), and offers better hemodynamics and durability^9^. The latest multi-center data from experienced centers demonstrated 10% in-hospital mortality and an additional 8% post-discharge mortality after median follow up of 10.8 years^10^. Despite benefits, Ross may risk both semilunar valves and require frequent re-intervention for right-ventricular-outflow-tract^11^. A meta-analysis of neonatal Ross demonstrated 24% in-hospital mortality and significant heterogeneity in existing cohorts^12^.

TVR is uncommon but necessary when truncal valve regurgitation (present in approximately one third of cases^13^) is unrepairable. A meta-analysis reports 49% early mortality and a 22% re-intervention rate in neonates and infants undergoing TVR^14^.

Intermediate term follow-up of AVR and Ross (e.g., median 6–8 years^15–17^) has been described. While small series discussed long-term outcomes up to 18 years^18^, large studies that discuss long-term outcomes come from single centers or few, highly experienced centers^10,19,20^, and even those studies are either limited to <15 years of follow-up or do not address infants separately. This highlights the need for multi-center research to inform clinical decisions^21^. Our study uses the Pediatric Cardiac Care Consortium (PCCC) Database to assess short- and long-term (25-year) outcomes, focusing on mortality and associated risk factors.

## Results

### Study overview and population characteristics

**Fig. 1:**
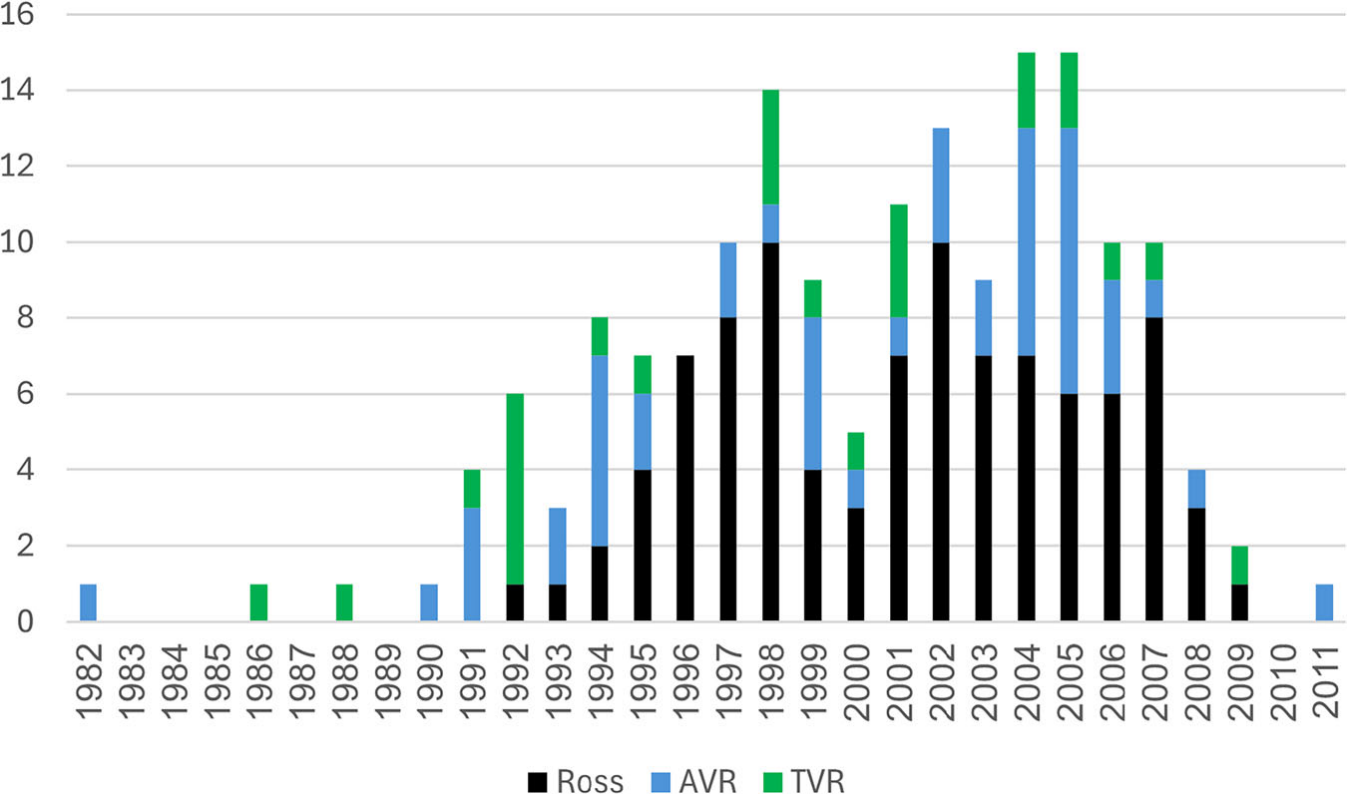
Annual counts. Annual number of systemic semilunar valve replacement operations in neonates and infants in US PCCC centers (1982–2011). Total number of operations:167. Ross (black), the Ross operation; AVR (blue) Aortic Valve Replacement, TVR (green) Truncal Valve Replacement, PCCC Pediatric Cardiac Care Consortium.

The PCCC query covered surgeries performed from December 15, 1982, to April 29, 2011, across 35 US centers. Systemic semilunar valve replacement procedures per center ranged from 1 to 15, with a median of 3 [Ross: median = 2 (range 0–8), AVR: median = 1 (range 0–5), TVR: median = 0 (range 0–4)]. Neonates undergoing systemic semilunar valve replacement represented 0.2% of all neonatal CHD surgeries, and infants 0.4%, totaling 0.36% of all neonatal/infant CHD surgeries. The annual procedure count ranged from 0 to 15 (Fig. 1).

Table 1 presents baseline characteristics for 167 patients: 64% male, 12% premature, 28% neonates ( ≤ 28 days), 3.6% <2.5 kg at surgery, and 15% with a genetic or chromosomal condition. Of these, 80 had identifiers for NDI tracking. Statistically significant differences between cohorts included race, length of stay, and surgical era. Patients with identifiers were operated on earlier and had higher in-hospital mortality (Supplementary Table 1). The most common primary diagnosis in the Ross and AVR groups was aortic stenosis, while all TVR patients had the diagnosis of Truncus Arteriosus (Supplementary Table 2).

**Table 1:** Baseline characteristics

1-A: whole cohort						*p* value
		Overall (*N* = 167)	Ross (*N* = 95)	AVR (*N* = 47)	TVR (*N* = 25)	
Sex	Female	60 (35.9%)	38 (40%)	9 (19.1%)	13 (52%)	0.010
	Male	107 (64.1%)	57 (60%)	38 (80.9%)	12 (48%)	
Age at surgery in days, median (IQR)		86 (22, 196)	97 (34-228)	66 (20-172)	34 (7-116)	0.008
Age group at surgery	Neonate	46 (28%)	22 (23.2%)	12 (25.5%)	12 (48%)	0.044
	Infant	121 (72%)	73 (76.8%)	35 (74.5%)	13 (52%)	
Race/ethnicity	Non-Hispanic White	49 (29.3%)	25 (26.3%)	11 (23.4%)	13 (52%)	0.197^a,b^
	Other race/ethnicity	6 (3.6%)	3 (3.2%)	3 (6.4%)	0 (0%)	
	Unknown	112 (67.1%)	67 (70.5%)	33 (70.2%)	12 (48%)	
Surgical era	Early (1982–2000)	77 (46.1%)	40 (42.1%)	22 (46.8%)	15 (60%)	0.278
	Late (2001–2011)	90 (53.9%)	55 (57.9%)	25 (53.2%)	10 (40%)	
Weight at surgery (Kg)	Median (IQR)	4.4 (3.5–6.1)	5.1 (3.8–6.3)	4.4 (3.5–6.4)	3.6 (2.6–4.3)	<0.001
	<2.5 Kg	6 (3.6%)	2 (2.1%)	2 (4.3%)	2 (8%)	0.356^a^
LOS (days)		12 (5, 32)	11 (6-32)	12 (4–29)	21 (0–36)	0.931
ECMO		18 (10.8%)	7 (7.4%)	9 (19.1%)	2 (8%)	0.092
Premature birth		20 (12%)	10 (10.5%)	4 (8.5%)	6 (24%)	0.125
Any chromosomal condition		6 (3.6%)	4 (4.2%)	2 (4.3%)	0 (0%)	0.728^a^
	Down Syndrome	1 (0.6%)	0 (0%)	1 (2.1%)	0 (0%)	
	Turner Syndrome	3 (1.8%)	3 (3.2%)	0 (0%)	0 (0%)	
	Other	2 (1.2%)	1 (1.1%)	1 (2.1%)	0 (0%)	
Other known genetic condition		19 (11.4%)	5 (5.3%)	5 (10.6%)	9 (36%)	<0.001
	DiGeorge Syndrome	18 (10.8%)	5 (5.3%)	4 (8.5%)	9 (36%)	<0.001
	Marfan Syndrome	0 (0%)	0 (0%)	0 (0%)	0 (0%)	
	Noonan Syndrome	0 (0%)	0 (0%)	0 (0%)	0 (0%)	
	Williams Syndrome	0 (0%)	0 (0%)	0 (0%)	0 (0%)	
	Other	1 (0.7%)	0 (0%)	1 (2.1%)	0 (0%)	

1-B: Cohort with Identifiers						
		Overall (*N* = 80)	Ross (*N* = 46)	AVR (*N* = 17)	TVR (*N* = 17)	
Sex	Female	31 (38.8%)	18 (39.1%)	5 (29.4%)	8 (47.1%)	0.571
	Male	49 (61.3%)	28 (60.9%)	12 (70.6%)	9 (52.9%)	

*AVR* Aortic Valve Replacement, *TVR* Truncal Valve Replacement, *LOS* Length of Stay, *ECMO* utilization of Extracorporeal membrane oxygenation post-operatively in the same hospitalization.

^a^ Fisher’s exact test.

^b^*p* value without unknown race.

### Mortality overview

**Fig. 2:**
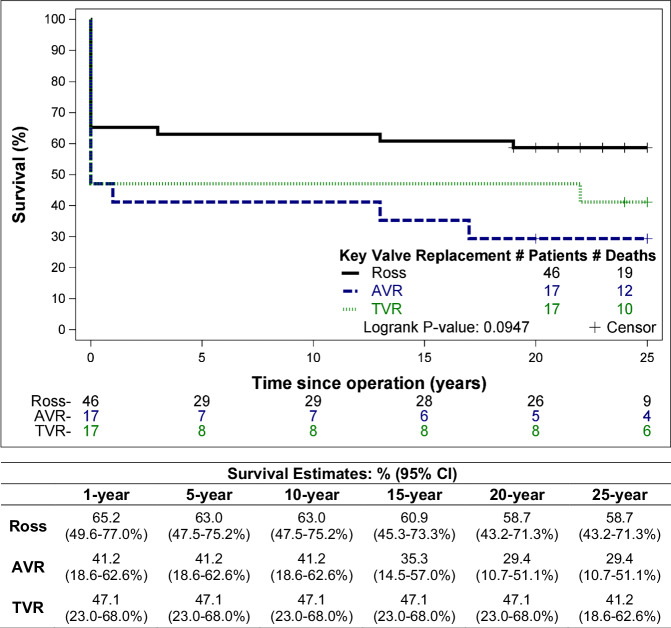
25-year survival-estimates. Kaplan-Meier curves estimating 25-year survival for neonates and infants undergoing systemic semilunar valve replacement with available identifiers for long-term follow up, stratified by operation type. Number at risk listed below graph, Survival estimates with confidence Intervals listed in table. *p* value calculated with Log Rank test. Ross (black line) the Ross procedure, AVR (blue dashed line) Aortic Valve Replacement, TVR (green dotted line) Truncal Valve Replacement, CI confidence interval. Plus sign indicates censored data.

Neonates and infants undergoing systemic semilunar valve replacement surgery had higher in-hospital mortality than those undergoing other cardiac surgeries (Table 2), with neonatal surgeries carrying the highest mortality, particularly for AVR. Long-term survival was followed for a median of 19 years (IQR 0–24 years), with 25-year survival estimates of 59% for Ross, 29% for AVR, and 41% for TVR (*p* = 0.095) (Fig. 2).

**Table 2:** In-hospital mortality for neonatal/infantile operations in US PCCC-centers,1982–2011

	Ross *N* (%)	AVR *N* (%)	TVR *N* (%)	Other *N* (%)	Total *N* (%)
Neonate	7/22 (31.8%)	8/12 (66.7%)	8/12 (66.7%)	3926/18638 (21.1%)	3949/18684 (21.1%)
Infant	15/73 (20.6%)	15/35 (42.9%)	5/13 (38.5%)	1959/27265 (7.2%)	1994/27386 (7.3%)
Total	22/95 (23.2%)	23/47 (48.9%)	13/25 (52.0%)	5885/45903 (12.8%)	5943/46070 (12.9%)

*PCCC* Pediatric Cardiac Care Consortium, *AVR* Aortic Valve Replacement, *TVR* Truncal Valve Replacement, *Othe*, non systemic semilunar valve replacement cardiac surgeries.

### Operation-specific outcomes (mortality and re-intervention)

**Fig. 3:**
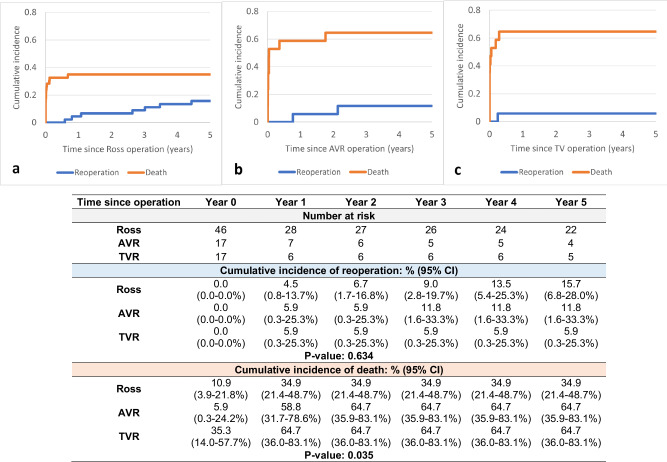
Competing risk analysis of death and re-intervention. Cumulative incidence of death and re-intervention 5-years post systemic semilunar valve replacement procedure in neonates and infants (by operation type). Significance determined by Gray’s test. **a** Ross (the Ross procedure): most attrition occurs in the first 1 year, but re-interventions continue to occur throughout the 5-year period. **b** AVR (Aortic Valve Replacement): most attrition and re-interventions occurred in the first 2 years.; **c** TVR (Truncal Valve Replacement): all deaths and re-interventions occurred in the first year.

Ross carried an overall in-hospital mortality rate of 23.2% (22/95); 31.8% in neonates and 20.6% in infants (Table 2). At 5 years, the cumulative incidence of re-intervention and death was 15.7% (95% CI; 6.8–28.0%) and 34.9% (95% CI; 21.4–48.7%), respectively, with most attrition occurring in the first 1 year, but re-interventions continued to occur throughout the 5-year period (Fig. 3).

AVR had an in-hospital mortality rate of 48.9% (23/47), 66.7% in neonates and 42.9% in infants (Table 2). At 5 years, the cumulative incidence of re-intervention and death was 11.8% (95% CI; 1.6–33.3%) and 64.7% (95% CI; 35.9–83.1%), respectively, with most attrition and re-interventions occurring in the first 2 years (Fig. 3).

TVR had an in-hospital mortality rate of 52.0% (13/25), 66.7% in neonates and 38.5% in infants (Table 2). At 5 years, the cumulative incidence of re-intervention and death was 5.9% (95% CI; 0.3–25.3%) and 64.7% (95% CI; 36.0–83.1%), respectively, all in the first year (Fig. 3).

### Factors associated with in-hospital mortality

Univariable analysis of in-hospital deaths showed increased mortality associated with AVR and TVR operations compared to Ross (OR 3.2 and 3.6, *p* = 0.002 and 0.006, respectively), neonatal age vs. infant (OR 2.5, *p* < 0.001), and earlier surgical era (1982–2000) vs. late (2001–2011) (OR 3.4, *p* < 0.001). Decreased mortality was associated with 1 kg increases in surgical weight (OR 0.67, *p* < 0.001) (Table 3). Multivariable analysis identified AVR had 3.5 times higher adjusted odds (aOR) of in-hospital mortality compared to Ross (*p* = 0.003). The early surgical era also saw 3.7 times higher adjusted odds of in-hospital mortality compared to the late era (*p* = 0.001). Meanwhile, surgical weight remained a protective factor (aOR 0.67, *p* < 0.001) (Table 3).

**Table 3:** Odds ratios (OR) with 95% confidence intervals (CI) for in-hospital deaths among the whole cohort

		Univariable				Multivariable		
			OR	95% CI	*p* value	aOR	95% CI	*p* value
Index operation	Ross		Ref.			Ref		
	AVR		3.18	1.51–6.70	0.002	3.47	1.51–7.93	0.003
	TVR		3.60	1.44–9.00	0.006	1.87	0.67–5.19	0.231
Sex	Female		1.14	0.59–2.21	0.694			
	Male		Ref.					
Age group at surgery	Neonate		2.46	1.22–4.94	<0.001			
	Infant		Ref					
Surgical weight (Kg)			0.67^a^	0.54–0.83	<0.001	0.67^a^	0.53–0.84	0.001
Surgical era	Early (1982–2000)		3.41	1.75–6.65	<0.001	3.67	1.76–7.68	0.001
	Late (2001–2011)		Ref			Ref.		
Chromosomal or known genetic condition	No		Ref					
	Yes		1.07	0.44–2.59	0.885			

Univariable and multivariable regression analyses.

*AVR* Aortic Valve Replacement, *TVR* Truncal Valve Replacement, *Ref* reference.

^a^OR for each 1 Kg increase. Stepwise backwards variable selection method utilized for multivariable logistic regression analysis with cut-off at *p* ≤ 0.20.

### Factors associated with long-term mortality

**Fig. 4:**
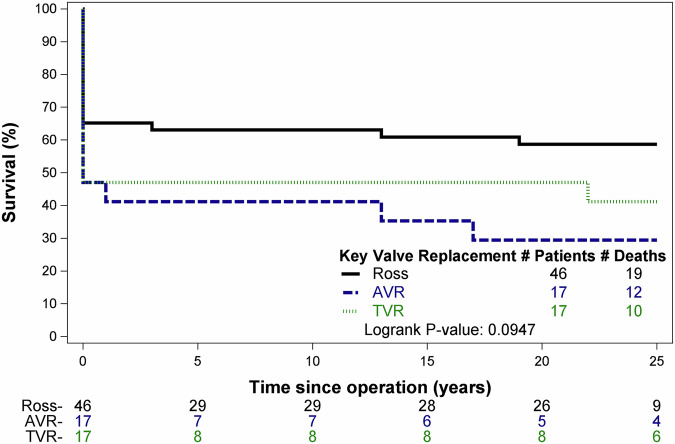
25-year survival-estimates for infants and neonates undergoing systemic semilunar valve replacement. Kaplan-Meier estimation of survival for systemic semilunar valve replacement with available identifiers for long-term follow up, stratified by operation type. Number at risk listed below graph. *p* value calculated with Log Rank test. Ross (black line) the Ross procedure, AVR (blue dashed line) Aortic Valve Replacement, TVR (green dotted line) Truncal Valve Replacement.

Among the cohort with available long-term follow-up, univariable (Fig. 4) analysis of cumulative mortality demonstrated AVR index (ref. Ross) [OR 3.4, *p* value 0.045] and neonatal age (ref. infant) [OR 3.92, *p* = 0.018] were associated with higher mortality, while higher surgical weight per 1 kg increase [OR 0.61, *p* = 0.002] was associated with lower mortality (Table 4). Multivariable analysis of cumulative mortality demonstrated AVR had 3.3 times higher adjusted odds of mortality compared to Ross (p = 0.075), and higher surgical weight per 1 kg increase was associated with lower cumulative mortality [aOR 0.59, *p* = 0.003] (Table 4).

**Table 4:** Odds ratios (OR) with 95% confidence intervals (CI) for cumulative deaths among the cohort with identifiers

			Univariable			Multivariable		
		OR		95% CI	*p* value	aOR	95% CI	*p* value
Index Operation	Ross	Ref.				Ref		
	AVR	3.41		1.03–11.29	0.045	3.28	0.89–12.10	0.075
	TV	2.03		0.66–6.29	0.220	0.84	0.23–3.07	0.787
Sex	Female	1.02		0.42–2.52	0.959			
	Male	Ref.						
Age group at surgery	Neonate	3.92		1.26–12.19	0.018			
	Infant	Ref.						
Surgical weight (Kg)		0.61^a^		0.45–0.84	0.002	0.59^a^	0.41–0.83	0.003
Surgical era	Early (1982–1996)	0.93		0.33–2.61	0.890			
	Late (1997–2003)	Ref.						
Chromosomal or Known genetic Condition	No	Ref						
	Yes	1.13		0.34-3.73	0.838			

Univariable and multivariable analyses.

*AVR* Aortic Valve Replacement, *TVR* Truncal Valve Replacement, *Ref* reference.

^a^OR for each 1 Kg increase. Stepwise backwards variable selection method utilized for multivariable logistic regression analysis with cut-off at *p* ≤ 0.20.

## Discussion

This analysis of a multi-institutional cohort of neonates and infants undergoing systemic semilunar valve replacement shows high in-hospital and long-term mortality associated with these procedures, highlighting the importance of longitudinal data for clinical practice and patient counseling.

The in-hospital mortality rates in our cohort (48.9% for AVR and 23.2% for Ross) were notably higher than previously reported rates. Prior studies have reported mortality rates of around 40% for AVR, 15.8% for Ross ± Konno^6^, and 10% for Ross^10^. The higher AVR mortality in our data may be due to the inclusion of an earlier surgical era (1982–2011 vs. 2000–2009 in Woods et al.). Similarly, the elevated Ross mortality may reflect the inclusion of centers with varying levels of experience, rather than only high-volume centers in Greenberg et al. For context, the Society of Thoracic Surgeons (STS) Congenital Heart Surgery Database reported a 14.7% mortality rate for STAT category 5 procedures across all ages—15% for neonates and 15.4% for infants—between January 2019 and December 2022^22^.

Our data show that Ross has better survival outcomes compared to AVR in neonates and infants. When compared to Ross, the AVR group had a nearly threefold increased adjusted odds of in-hospital and cumulative mortality. Along the same lines, post-operative mechanical support was most utilized in the AVR group (19%) in our cohort, and the 25-year estimated survival for Ross was 59% vs 29% for AVR. This difference is observed in neonates and infants and was not captured in previous work on ages 1-6 years^19^. One of the reasons for this improved early outcome for Ross in our cohort could be that the median age and weight was higher in the Ross group than that of AVR and TVR. Mortality generally stabilized after 1 year of surgery for all surgeries, but delayed AVR mortality was observed more than a decade later. A similar pattern has been reported in other ages^19^. The difference in long-term mortality between Ross and AVR may be related to the somatic growth of the Ross autograft and the ability to perform re-interventions on the pulmonary valve in the catheterization lab. As expected, RVOT re-intervention was the most common in our Ross cohort (occurring in 25/41 of all re-interventions in the first 5 years) (Supplementary Tables 3 and 4).

Higher surgical weight was strongly associated with better outcomes in both the short- and long-term. Increased weight enhances the technical feasibility of procedures, and even small increases in weight were significantly linked to reduced early and late mortality. Similarly, neonatal age at index operation was associated with a higher likelihood of in-hospital mortality compared to infancy. This better survival of infants compared to neonates could be explained by the protective effect of higher weight; however, selection bias may also be at play (i.e., neonates may be sicker at presentation).

The later surgical era was associated with lower in-hospital mortality in our cohort. This has been shown in prior works describing other age groups and other procedures^23–25^. We believe that this era effect is multifactorial and is likely due to a combination of improved surgical expertise as well as cardiopulmonary bypass, perfusion, and intensive care techniques. There may be an additional bias given the slight predominance of Ross over AVR in the later era (40 Ross and 22 AVR in the early era, 55 Ross and 25 AVR in the later era).

Like prior data^19^, our AVR group was predominantly male (81%) and so was the Ross group (60%), highlighting that congenital aortic valve stenosis is a male-predominant disease^26^. While 15% of our cohort had a detected chromosomal or genetic diagnosis (somewhat matching existing literature for older age groups), these numbers may be an underestimate given changing practices and methods of genetic screening during the study period. Nonetheless, our data show that 22q11 deletion was more common in the Truncus Arteriosus group.

Re-intervention analyses may be biased toward the null due to incomplete long-term follow-up and attrition. Patients may relocate and receive care at non–PCCC-participating centers. This limitation is particularly relevant for Ross procedure, in which pulmonary conduit re-interventions often occur earlier, whereas neo-aortic root or valve re-interventions related to autograft dilation or neo-aortic insufficiency tend to occur later. As a result, accurate comparisons of re-intervention rates (e.g., pulmonary vs. neo-aortic re-intervention following Ross, or re-intervention following Ross vs. AVR) cannot be reliably calculated within the available follow-up window (Supplementary Tables 3 and 4).

In summary, neonates and infants needing systemic semilunar valve replacement have high short- and long-term mortality. To address the unique challenge of truncal valve disease where Ross is not an option, newer strategies of delivering growing valve implants, namely Partial Heart Transplantation (PHT)^27^ are being studied^28,29^ and showing promising short-term results^30^. PHT has also been reported to be used as a “living Ross” whereby the pulmonary valve is replaced by a PHT to avoid future re-interventions^31^. Another technique was described by Ozaki et al in 2011 that involves creation of a new valve from pericardium and has been reported to have good short-term outcomes^32^. While these newer techniques may theoretically help improve long-term mortality and reduce re-intervention rates, long-term data remains to be seen^33^.

This study is not without limitations. Obtaining long-term outcomes inevitably involves using historical data that may not fully reflect current practice; nevertheless, such data remain valuable, especially since Ross and AVR continue to be the primary surgical options. In addition, our study did not account for additional procedures at the time of Ross (Konno, mitral valve operation, aortic arch repair), although more recent and granular data did not show a significant difference in operative details (i.e., concomitant Konno, arch surgery, mitral surgery, EFE resection, and VSD closure) between survivors and non-survivors^10^. The analysis of surgical era effect per index operation was not feasible due to the small numbers in each individual subgroup, which also limited the feasibility of propensity score analysis. Additionally, we did not have the data available to look at the outcomes of the different types of AVR (homograft vs bioprosthetic). The significant differences between the long-term follow-up group and the whole cohort may also limit generalizability of our findings. Analysis of factors associated with mortality for each index operation was limited by low statistical power and lack of granular details in database studies. Of note, this study focused on valve replacement and did not account for any prior valve repairs, which are generally the preferred initial approach when feasible to delay timing of Ross or valve replacement^33^.

## Methods

### Study description

We conducted a retrospective cohort study using the PCCC registry, a large US-based database for pediatric cardiovascular interventions^34,35^, similar to our previous work about pulmonary valve replacement^36^. The study was approved by the Emory University IRB (approval number 00080706, issued on 6/30/2015, with the last annual review dated 11/8/2024) and granted an IRB waiver by the University of Arkansas for Medical Sciences since no patient identifying data was shared (number 297565, 8/15/2024). The research involves minimal risk to subjects, such as reviewing medical records for limited, non-sensitive information. No patient-identifying data is presented. Informed consent was waived.

### Patient population

We queried the PCCC registry for all patients who underwent Ross, AVR, or TVR procedure before 1 year of age in a US PCCC center between 1982 and 2011 (*n* = 167). Patients were identified as AVR if they had a diagnostic code for AVR and as Ross if they had codes for AVR and PVR in the same operation or AVR with a Konno procedure. TVR patients included those with a TVR procedural code or an AVR procedure with a concurrent diagnosis code of Truncus arteriosus.

A subgroup of patients was selected for intermediate- and long-term outcome analysis if they had available identifiers: first name, state of residence, sex, US residency, and a birthdate before April 15, 2003, the day that HIPAA regulations changed (*n* = 80).

The US National Death Index (NDI) provided data on deaths through 2022. The linkage between the PCCC and NDI has been shown to achieve 88.1% sensitivity and 99.8% specificity for patients with full identifiers^24,37^.

Re-interventions were defined as any relevant operation or catheter-based re-intervention that occurred up to 5 years after the index operation, including those that occurred within the same hospital admission. Analysis beyond 5 years was not meaningful due to limited numbers and possible attrition as patients may move from their original center of care. Relevant re-interventions included any aortic valve surgery, aortic valve repair, AVR, aortic valve balloon dilation, supravalvar aortic stenosis surgery, Konno procedure, aortic root replacement, truncal valve procedures, and all pulmonary valve surgeries and catheterization procedures.

### Data analysis

Patient characteristics included sex, race/ethnicity (if available), prematurity, chromosomal and genetic disorders, age, and weight at operation, surgical era, hospital discharge status, post-operative extracorporeal membrane oxygenation (ECMO) use, and length of stay (LOS). Surgical eras were dichotomized based on sample size: early (1982–2000 for in-hospital mortality; 1982–1996 for long-term mortality) and late (2001–2011 for in-hospital mortality; 1997–2003 for long-term mortality).

Continuous variables were reported as medians with interquartile ranges (IQR) and compared using the Kruskal-Wallis test. Categorical variables were presented as frequencies and compared using the Chi-square or Fisher exact test for cell counts <5.

Logistic regression analyzed factors associated with in-hospital mortality by operation type. Backwards stepwise selection identified variables with *p* < 0.20 for multivariable modeling. Covariates assessed included age, sex, weight, surgical era, and genetic diagnoses.

For patients with available identifiers, Kaplan-Meier plots estimated 25-year survival by operation type, with significance assessed via log-rank tests. Competing risk analysis evaluated 5-year re-intervention and mortality rates, with significance determined by Gray’s test.

Statistical significance was set at *p* < 0.05. Analyses were conducted using SAS 9.4 (SAS Institute Inc., Cary, NC, USA).

## Supplementary information

Supplementary information

## Data Availability

The datasets analyzed during the current study are available in the Pediatric Cardiac Care Consortium (PCCC) repository, https://pcccweb.com/.

## Code availability

The variables were produced using standard SAS 9.4 software. The analysis plan and codes are available upon request.
