# Supplementary material for: Short- and long-term outcomes of systemic semilunar valve replacement in neonates and infants
Source: NPJ Cardiovasc Health. 2026 Mar 23;3:12. doi: 10.1038/s44325-026-00109-6 (PMC13009365; doi:10.1038/s44325-026-00109-6)
Supplement: Supplementary file 1 — Supplementary information [file 44325_2026_109_MOESM1_ESM.pdf]

Supplemental Table 1: Comparison of Whole Cohort with and without available Identifiers for intermediate- and long-term outcome analysis					
		Overall (N=167)	With Identifiers (N=80)	Without identifiers (N=87)	p-value
Sex	Female	60 (35.9%)	31 (38.8%)	29 (33.3%)	0.466
	Male	107 (64.1%)	49 (61.3%)	58 (66.7%)	
Age at surgery (days)	Median (IQR)	86 (22-196)	94 (28-198)	73 (20-169)	0.516
Age group at surgery	Neonate	46 (27.5%)	20 (25.0%)	26 (29.9%)	0.480
	Infant	121 (72.5%)	60 (75.0%)	61 (70.1%)	
Race/ethnicity	White	49 (29.3%)	40 (50.0%)	9 (10.3%)	0.023[a,b]
	Other	6 (3.6%)	2 (2.5%)	4 (4.6%)	
	Unknown	112 (67.1%)	38 (47.5%)	74 (85.1%)	
Surgical era[c]	Early (1982-2000)	77 (46.1%)	61 (76.3%)	16 (18.4%)	<0.001
	Late (2001-2011)	90 (53.9%)	19 (23.8%)	71 (81.6%)	
Surgical era[d]	Early (1982-1996)	39 (23.4%)	35 (43.8%)	4 (4.6%)	<0.001
	Late (1997-2003)	128 (76.6%)	45 (56.3%)	83 (95.4%)	
Weight at surgery (kg)	Median (IQR)	4.4 (3.5, 6.1)	4.4 (3.6, 6.1)	4.3 (3.5, 6.2)	0.977
	<2.5 kg	6 (3.6%)	2 (2.5%)	4 (4.6%)	0.684
LOS (days)	Median (IQR)	12 (5, 32)	9 (4, 21.5)	16.0 (7.0, 56.0)	0.006
ECMO		18 (10.8%)	10 (12.5%)	8 (9.2%)	0.491
In-hospital mortality		58 (34.7%)	34 (42.5%)	24 (27.6%)	0.043
Premature birth		20 (12.0%)	12 (15.0%)	8 (9.2%)	0.248
Any chromosomal disorder		6 (3.6%)	3 (3.8%)	3 (3.4%)	1.000[a]
	Down's Syndrome	1 (0.6%)	0 (0.00%)	1 (1.1%)	
	Turner Syndrome	3 (1.8%)	2 (2.5%)	1 (1.1%)	
	Other	2 (1.2%)	1 (1.3%)	1 (1.2%)	
Any genetic disorder		19 (11.4%)	10 (12.5%)	9 (10.3%)	0.661
	DiGeorge Syndrome	18 (10.8%)	10 (12.5%)	8 (9.2%)	0.491[a]
	Marfans Syndrome	0 (0.00%)	0 (0.00%)	0 (0.00%)	-
	Noonans Syndrome	0 (0.00%)	0 (0.00%)	0 (0.00%)	-
	Williams Syndrome	0 (0.00%)	0 (0.00%)	0 (0.00%)	-
	Other	1 (0.7%)	0 (0.00%)	1 (1.2%)	

LOS, Length of Stay; ECMO, utilization of Extracorporeal membrane oxygenation post-operatively in the same hospitalization; [a] Fisher's exact test; [b] p-value without unknown race; [c] Dichotomization for whole cohort; [d] Dichotomization for cohort with identifiers.

Supplemental Table 2: Primary Diagnosis for each valve replacement (N, %)

	Ross (N = 95)		AVR (N= 47)
1	Aortic stenosis (48, 50.5%)	1	Aortic stenosis (24, 51%)
2	Coarctation with any of ASD, VSD, AS (24, 25.3%)	2	Interrupted aortic arch (5, 10.6%)
3	Interrupted aortic arch (10, 10.5%)	3	Single ventricle dominant RV (4, 8.5%)
	Others (13, 13.7%)	4	Coarctation of the aorta (3, 6.4%)
			Others (11, 23.4%)

	TVR (N=25)
1	Truncus Arteriosus (25, 100%)

AVR, Aortic Valve Replacement; TVR, Truncal Valve Replacement; ASD, Atrial Septal Defect; VSD, Ventricular Septal Defect; AS, Aortic Stenosis; RV, Right Ventricle.

Supplemental Table 3: Description of procedures and re-interventions among neonates with valve replacements in US PCCC centers between 1982 and 2011				
	Index surgery for semilunar valve replacement			
	Ross n (%)	AVR n (%)	TVR n (%)	Total N (%)
Total # patients	21	12	12	45
Times reoperated				
0	18 (85.7)	9 (75.0)	10 (83.3)	37 (82.2)
1	2 (9.5)	3 (25.0)	2 (16.7)	7 (15.6)
2	1 (4.8)	0 (0.0)	0 (0.0)	1 (2.2)
Total # re-interventions[a]	4	3	2	9
Age at re-intervention				
Neonate	0 (0.0)	0 (0.0)	0 (0.0)	0 (0.0)
Infant	2 (50.0)	3 (100.0)	2 (100.0)	7 (77.8)
Toddler	1 (25.0)	0 (0.0)	0 (0.0)	1 (11.1)
4 to 9 years	1 (25.0)	0 (0.0)	0 (0.0)	1 (11.1)
10 to 20 years	0 (0.0)	0 (0.0)	0 (0.0)	0 (0.0)
Total # re-intervention procedures[b]	5	3	3	11
Type of re-intervention procedure				
Aortic valve replacement	0 (0.0)	3 (100.0)	2 (66.7)	5 (45.5)
Prosthetic valve (bio or mechanical)	0	2	2	4
Ross	0	1	0	1
Pulmonary valve surgery or catheterization	5 (100.0)	0 (0.0)	1 (33.3)	6 (54.5)

AVR, Aortic Valve Replacement; TVR, Truncal Valve Replacement, All % are by columns, [a] N of re-interventions on unique surgical dates post-systemic semilunar valve replacement, [b] N of all related surgical codes completed post-semilunar valve replacements. Note some patients had more than one procedure during the re-intervention.

Supplemental Table 4: Description of procedures and re-interventions among infants with valve replacements in US PCCC centers between 1982 and 2011				
	Index surgery for semilunar valve replacement			
	Ross<br>n (%)	AVR<br>n (%)	TVR<br>n (%)	Total<br>N (%)
Total # patients	74	35	13	122
Times reoperated				
0	55 (74.3)	26 (74.3)	12 (92.3)	93 (76.2)
1	14 (18.9)	9 (25.7)	1 (7.7)	24 (19.7)
2	5 (6.8)	0 (0.0)	0 (0.0)	5 (4.1)
Total # re-interventions[a]	24	9	1	34
Age at re-intervention				
Neonate	0 (0.0)	0 (0.0)	0 (0.0)	0 (0.0)
Infant	0 (0.0)	2 (22.2)	0 (0.0)	2 (5.9)
Toddler	11 (45.8)	5 (55.6)	0 (0.0)	16 (47.0)
4 to 9 years	11 (45.8)	1 (11.1)	0 (0.0)	12 (35.3)
10 to 20 years	2 (8.3)	1 (11.1)	1 (100.0)	4 (11.8)
Total # re-intervention procedures[b]	36	11	2	49
Type of re-intervention procedure				
Aortic valve replacement	6 (16.7)	8 (72.7)	1 (50.0)	15 (30.6)
Prosthetic valve (bio or mechanical)	6	4	1	11
Ross	0	4	0	4
Other aortic site surgery	10 (27.8)	2 (18.2)	0 (0.0)	12 (24.5)
Konno procedure	3	1	0	4
Supravalvar aortic stenosis surgery	1	0	0	1
Pulmonary valve surgery or catheterization	20 (55.6)	1 (9.1)	1 (50.0)	22 (44.9)

AVR, Aortic Valve Replacement; TVR, Truncal Valve Replacement, All % are by columns, [a] N of re-interventions on unique surgical dates post-systemic semilunar valve replacement, [b] N of all related surgical codes completed post-systemic semilunar valve replacements. Note some patients had more than one procedure during the re-intervention.
